# Supplementary material for: From sequence to enzyme mechanism using multi-label machine learning
Source: BMC Bioinformatics. 2014 May 19;15:150. doi: 10.1186/1471-2105-15-150 (PMC4229970; doi:10.1186/1471-2105-15-150)
Supplement: Additional file 2 — Java code of ml2db. Additional file ml2db_code.tar.gz contains the Java source code to run the multi-label machine learning experiments and save the results to database. The code’s Javadoc is included. [file 1471-2105-15-150-S2.zip › additional file 2/ml2db/ml2db/doc/mulan/classifier/transformation/class-use/PrunedSets.Strategy.html]

Uses of Class mulan.classifier.transformation.PrunedSets.Strategy


JavaScript is disabled on your browser.


- Overview
- Package
- Class
- Use
- Tree
- Deprecated
- Index
- Help

- Prev
- Next

- Frames
- No Frames

- All Classes

## Uses of Class mulan.classifier.transformation.PrunedSets.Strategy

- Packages that use PrunedSets.Strategy

  | Package | Description |
  | --- | --- |
  |  |  |
  | --- | --- |
  | mulan.classifier.transformation |  |
- - ### Uses of PrunedSets.Strategy in mulan.classifier.transformation

    Methods in mulan.classifier.transformation that return PrunedSets.Strategy

    | Modifier and Type | Method and Description |
    | --- | --- |
    |  |  |
    | --- | --- |
    | `static PrunedSets.Strategy` | PrunedSets.Strategy.`valueOf(java.lang.String name)` Returns the enum constant of this type with the specified name. |
    | `static PrunedSets.Strategy[]` | PrunedSets.Strategy.`values()` Returns an array containing the constants of this enum type, in the order they are declared. |

    Constructors in mulan.classifier.transformation with parameters of type PrunedSets.Strategy

    Constructor and Description

    `EnsembleOfPrunedSets(double aPercentage,
    int aNumOfModels,
    double aThreshold,
    int aP,
    PrunedSets.Strategy aStrategy,
    int aB,
    weka.classifiers.Classifier baselearner)`

    `PrunedSets(weka.classifiers.Classifier classifier,
    int aP,
    PrunedSets.Strategy aStrategy,
    int aB)`

    Constructor that initializes learner with base algorithm, parameter p
    and strategy for processing infrequent labelsets

- Overview
- Package
- Class
- Use
- Tree
- Deprecated
- Index
- Help

- Prev
- Next

- Frames
- No Frames

- All Classes
